# Understanding Resident, Family, and Staff Safety Priorities to Guide Development of an Engagement Toolkit

**DOI:** 10.1093/geroni/igab046.1333

**Published:** 2021-12-17

**Authors:** Victoria Bartoldus, Cloie Chiong, Tabitha Linville, Stephanie Palmertree, Anna Beeber, Youngmin Cho

**Affiliations:** 1 University of North Carolina at Chapel Hill, Chapel Hill, North Carolina, United States; 2 University of North Carolina at Chapel Hill Gillings school of Global Public Health, Chapel Hill, North Carolina, United States; 3 University of North Carolina at Chapel Hill School of Nursing, Chapel Hill, North Carolina, United States; 4 University of North Carolina at Chapel Hill, UNC Chapel Hill, North Carolina, United States

## Abstract

Resident and family engagement (the desire, ability, and activation as a partner in care) is a necessary component of keeping assisted living (AL) residents safe. Barriers to engagement include differing priorities between the resident/family and staff. This presentation outlines the results of a content analysis of qualitative interviews with 105 AL staff, residents, and family members, in which we examined AL stakeholder priorities for safety. Qualitative interviews were analyzed to first identify safety priorities by stakeholder type (staff, resident, and families), and then compared across stakeholder group. Stakeholder-specific safety priorities were identified, including infection management (COVID-19 and others), medications errors, falls, elopement, lack of AL resources/staffing, conflict, adverse events, nutrition, physical hazards, building security, chemical agents, fire/natural disasters, and abuse/neglect – the importance of these priorities vary by stakeholder type. Presentation discussion will include implications for future intervention to address the top safety problems in AL.

